# Supplementary material for: Universal and efficient extraction of lithium for lithium-ion battery recycling using mechanochemistry
Source: Commun Chem. 2023 Mar 28;6:49. doi: 10.1038/s42004-023-00844-2 (PMC10049983; doi:10.1038/s42004-023-00844-2)
Supplement: Supplementary file 2 — Supplementary Information [file 42004_2023_844_MOESM2_ESM.pdf]

Supplementary information to the manuscript

Universal and efficient extraction of lithium for lithium-ion battery recycling using mechanochemistry.

Oleksandr Dolotko[1,2*], Niclas Gehrke[1], Triantafillia Malliaridou[1], Raphael Sieweck[1], Laura Herrmann[1,3], Bettina Hunzinger[1], Michael Knapp[1], Helmut Ehrenberg[1,2]

[1] Karlsruhe Institute of Technology (KIT), Institute for Applied Materials-Energy Storage Systems (IAM-ESS), Hermann-von-Helmholtz-Platz 1, D-76344 Eggenstein-Leopoldshafen, Karlsruhe, Germany

[2] Helmholtz-Institute Ulm for Electrochemical Energy Storage (HIU), P.O. Box 3640, D-76021 Karlsruhe, Germany

[3] EnBW Energie Baden-Württemberg AG, Durlacher Allee 93, 76131 Karlsruhe, Germany

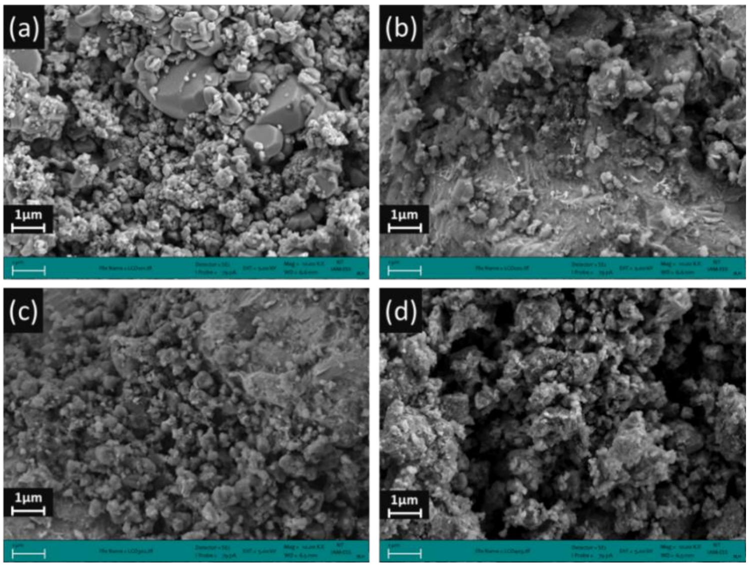

Fig.S1. SEM image of the LCO powder-(a) ball milled with Al foil for 0.5 hours - (b), 1 hour - (c), 3 hours - (d).

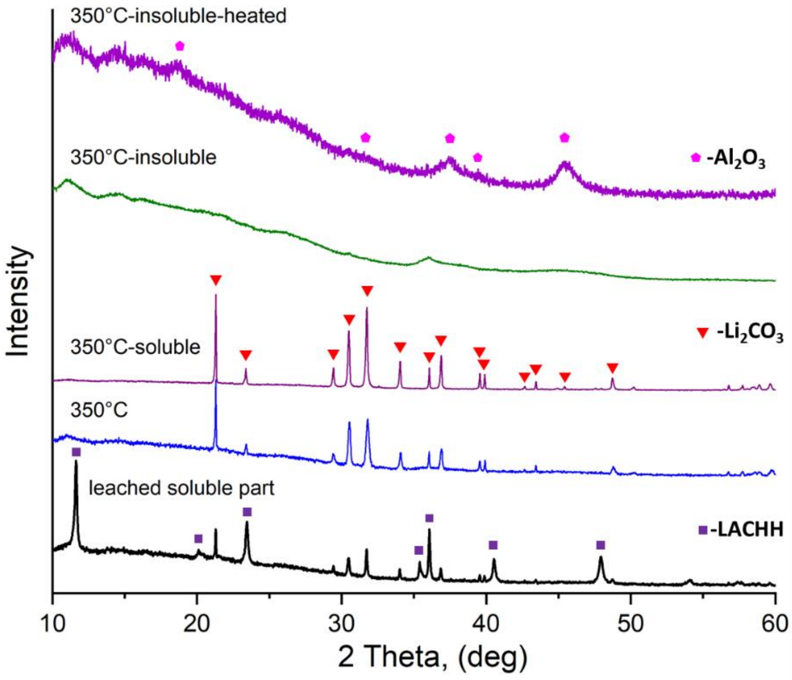

Fig. S2. XRD patterns of the products during the purification process. The most intensive Bragg reflections of intermediate and final products are marked for analysis.

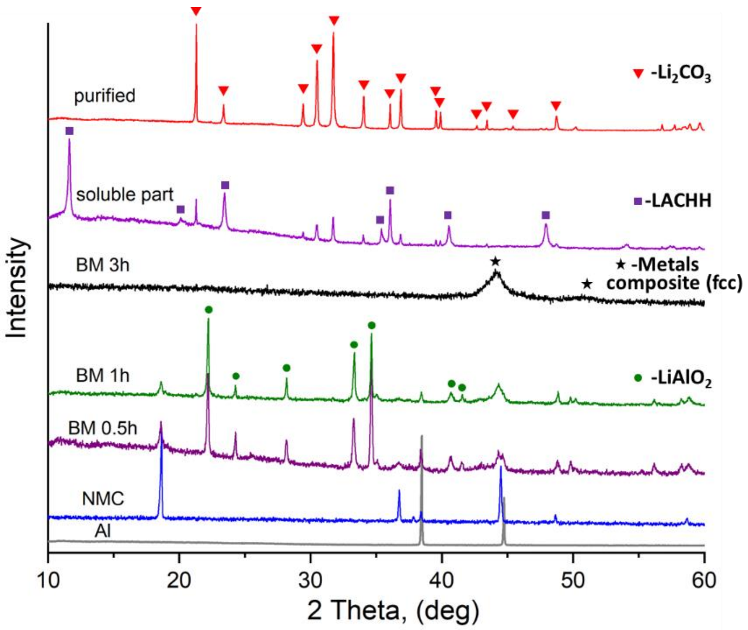

Fig. S3. XRD patterns of the 1:1 molar mixture of $Li(Ni_{0.33}Mn_{0.33}Co_{0.33})O_2$ and Al measured after different ball milling times in a SPEX mill. XRD patterns of starting materials NMC and Al are presented for comparison. The most intensive Bragg reflections of intermediate and final products are marked for analysis.

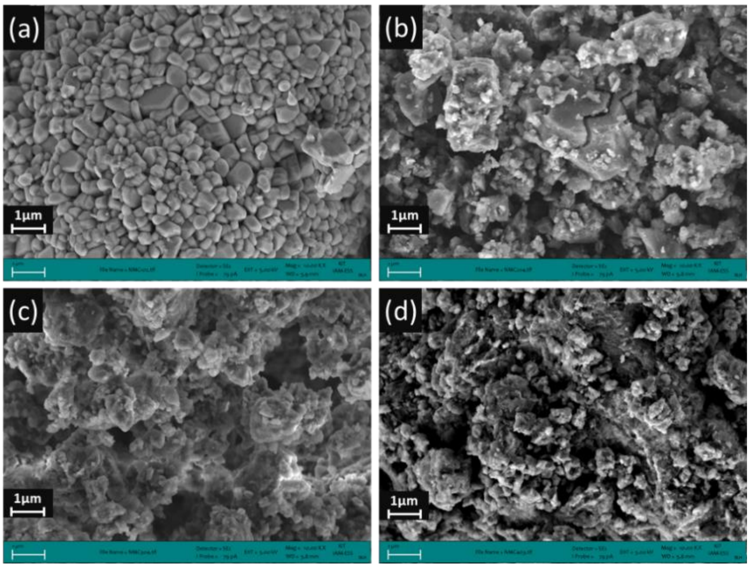

Fig. S4. SEM image of the NMC powder-(a) ball milled with Al foil for 0.5 hours - (b), 1 hour - (c), 3 hours - (d).

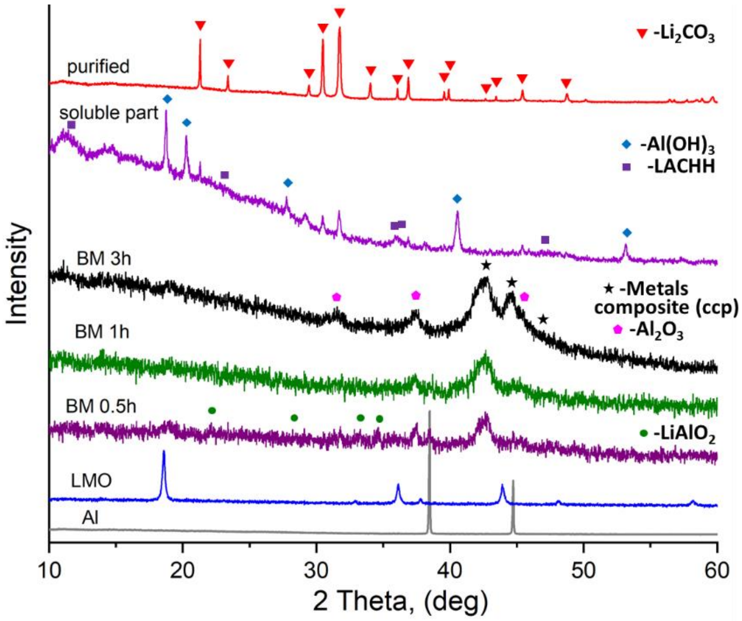

Fig. S5. XRD patterns of the 1:2.33 molar mixture of $LiMn_2O_4$ and Al, measured after different ball milling times in a SPEX mill. XRD patterns of starting materials LMO and Al are presented for comparison. The most intensive Bragg reflections of intermediate and final products are marked for analysis.

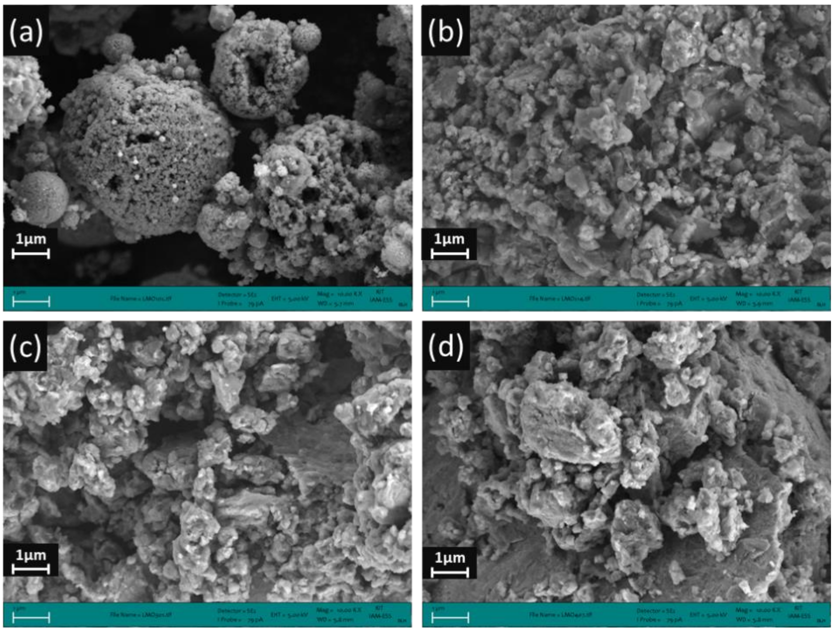

Fig. S6. SEM image of the LMO powder-(a) ball milled with Al foil for 0.5 hours - (b), 1 hour - (c), 3 hours - (d).

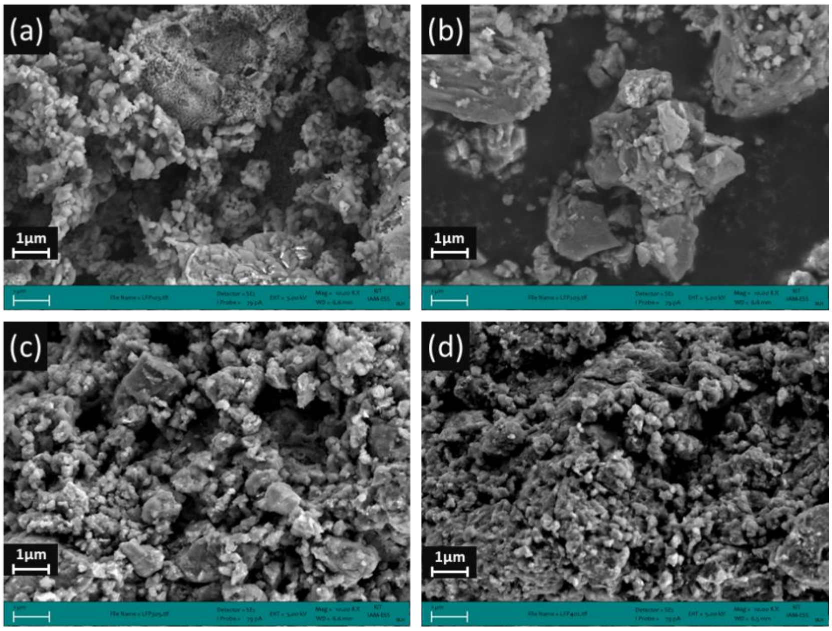

Fig. S7. SEM image of the LFP powder-(a) ball milled with Al foil for 0.5 hours - (b), 1 hour - (c), 3 hours - (d).

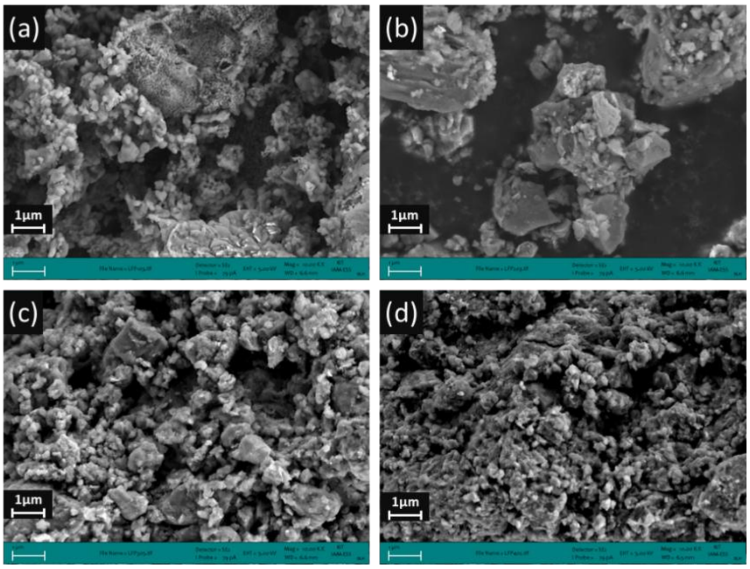

Fig. S8. SEM image of the mixture of LCO, NMC, LMO and LFP cathodes - (a) ball milled with Al foil for 0.5 hours - (b), 1 hour - (c), 3 hours - (d).

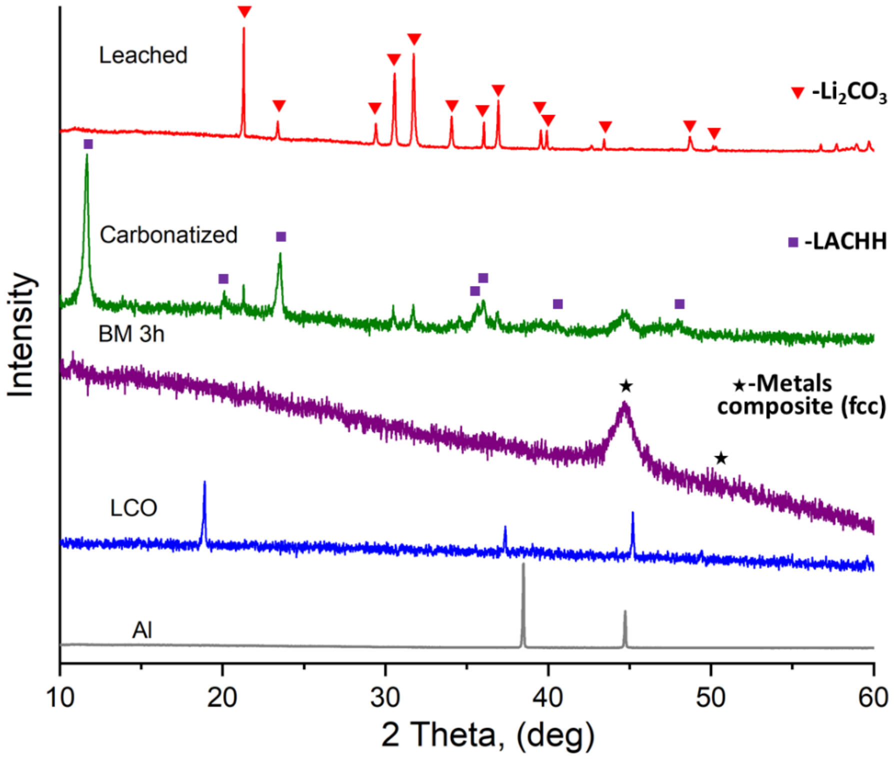

Fig. S9. XRD patterns of the 1:1 molar mixture of $LiCoO_2$ and Al, measured after different steps of process 2. XRD patterns of starting materials LCO and Al are presented for comparison. The most intensive Bragg reflections of intermediate and final products are marked for analysis.

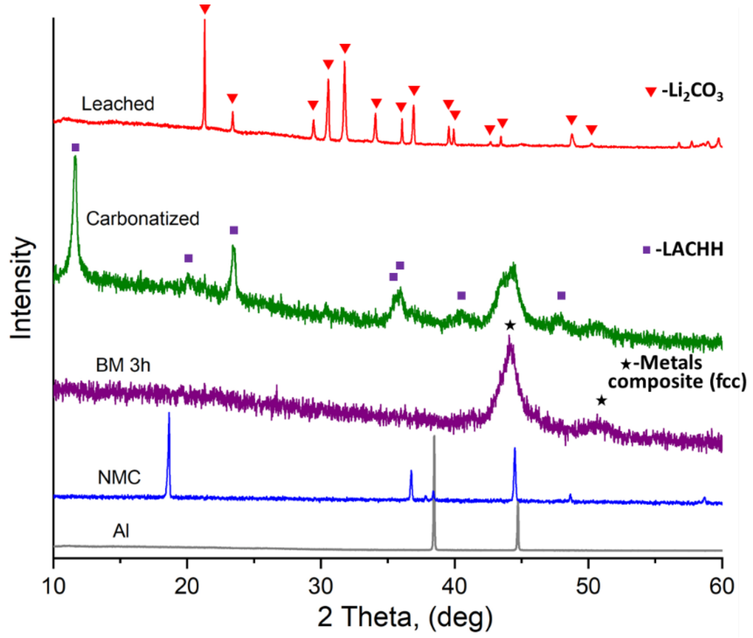

Fig. S10. XRD patterns of the 1:1 molar mixture of $Li(Ni_{0.33}Mn_{0.33}Co_{0.33})O_2$ and Al, measured after different steps of process 2. XRD patterns of starting materials NMC and Al are presented for comparison. The most intensive Bragg reflections of intermediate and final products are marked for analysis.

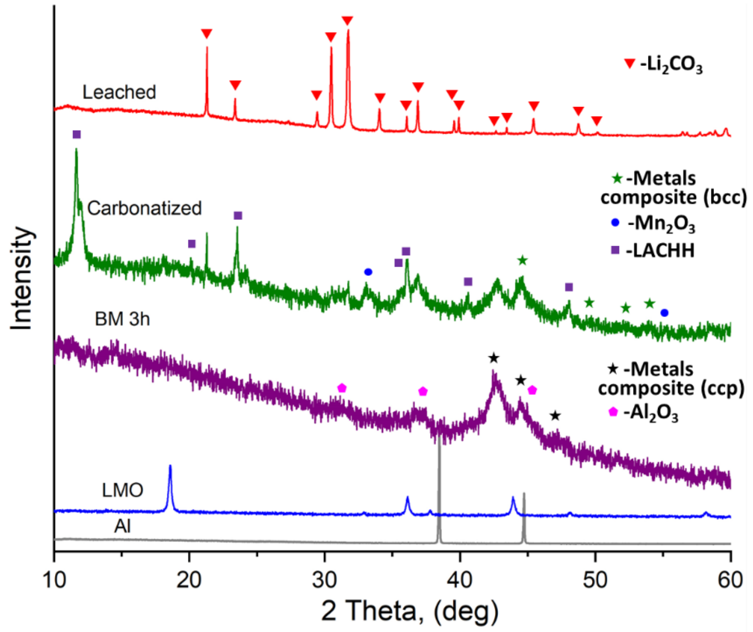

Fig. S11. XRD patterns of the 1:2.33 molar mixture of $LiMn_2O_4$ and Al, measured after different steps of process 2. XRD patterns of starting materials LMO and Al are presented for comparison. The most intensive Bragg reflections of intermediate and final products are marked for analysis.

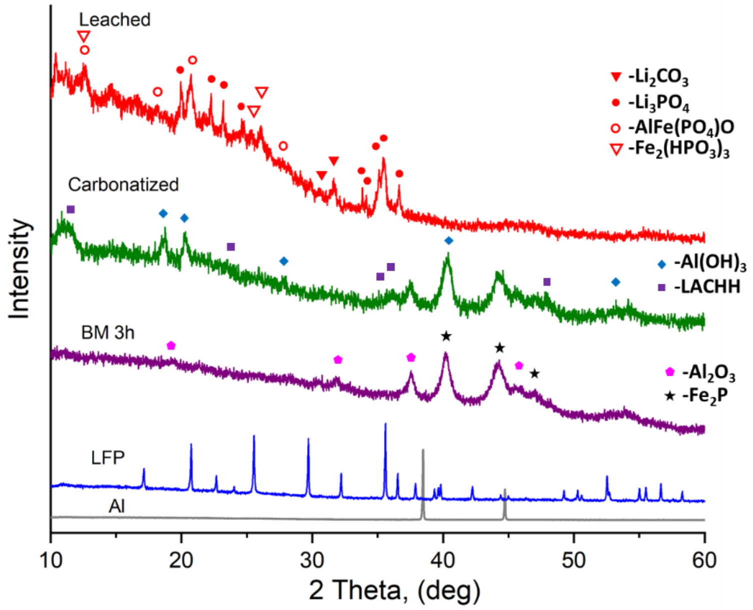

Fig. S12. XRD patterns of the 1:3 molar mixture of $LiFePO_4$ and Al, measured after different steps of Process 2. XRD patterns of starting materials LFP and Al are presented for comparison. The most intensive Bragg reflections of intermediate and final products are marked for analysis.

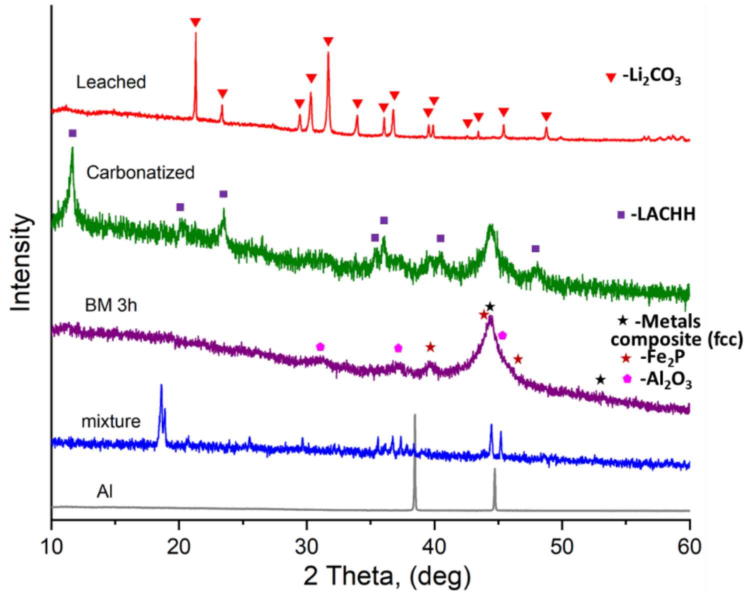

Fig. S13. XRD patterns of the mixture of LCO, NMC, LMO and LFP cathodes with Al, measured after different steps of process 2. XRD patterns of starting materials - cathode physical mixture and Al, are presented for comparison. The most intensive Bragg reflections of intermediate and final products are marked for analysis.

Supplementary Methods.

Chemical Analysis of the products.

For sample preparation, Li_2CO_3 materials were dissolved in bidistilled water.

The purity of lithium carbonate, as well as the content of impurities, was determined based on ICP-OES analyses using equations S1 and S2. The purity of lithium carbonate was determined by the ratio of the mass of lithium carbonate ($m_{Li_2CO_3}$) to the sum of the mass of lithium carbonate and the mass of any impurities present (m_i), such as Al, Co, Ni, Mn, Fe, and P.

$$Purity\ (Li_2CO_3)\ [\%] = \frac{m_{Li_2CO_3}}{m_{Li_2CO_3}+m_{Al}+m_{Co}+m_{Ni}+m_{Mn}+m_{Fe}+m_P} \cdot 100 \qquad \text{(Eq. S1)}$$

The content of impurity of element i, can be expressed as the ratio of the mass of this element to the mass of lithium carbonate.

$$Impurity\ (i)[\mu g/mg\ (Li_2CO_3)] = \frac{m_i}{m_{Li_2CO_3}} \qquad \text{(Eq. S2)}$$

The results of these calculations are shown in Table 1.
